# A bibliometric analysis of researches on flap endonuclease 1 from 2005 to 2019

**DOI:** 10.1186/s12885-021-08101-2

**Published:** 2021-04-07

**Authors:** Qiaochu Wei, Jiming Shen, Dongni Wang, Xu Han, Jing Shi, Lei Zhao, Yuee Teng

**Affiliations:** Department of Medical Oncology, The First Hospital of China Medical University, Shenyang, 110001 China

**Keywords:** Flap endonuclease 1, Bibliometrics, Citespace, Cancer

## Abstract

**Background:**

Flap endonuclease 1 (FEN1) is a structure-specific nuclease that plays a role in a variety of DNA metabolism processes. FEN1 is important for maintaining genomic stability and regulating cell growth and development. It is associated with the occurrence and development of several diseases, especially cancers. There is a lack of systematic bibliometric analyses focusing on research trends and knowledge structures related to FEN1.

**Purpose:**

To analyze hotspots, the current state and research frontiers performed for FEN1 over the past 15 years.

**Methods:**

Publications were retrieved from the Web of Science Core Collection (WoSCC) database, analyzing publication dates ranging from 2005 to 2019. VOSviewer1.6.15 and Citespace5.7 R1 were used to perform a bibliometric analysis in terms of countries, institutions, authors, journals and research areas related to FEN1. A total of 421 publications were included in this analysis.

**Results:**

Our findings indicated that FEN1 has received more attention and interest from researchers in the past 15 years. Institutes in the United States, specifically the Beckman Research Institute of City of Hope published the most research related to FEN1. Shen BH, Zheng L and Bambara Ra were the most active researchers investigating this endonuclease and most of this research was published in the Journal of Biological Chemistry. The main scientific areas of FEN1 were related to biochemistry, molecular biology, cell biology, genetics and oncology. Research hotspots included biological activities, DNA metabolism mechanisms, protein-protein interactions and gene mutations. Research frontiers included oxidative stress, phosphorylation and tumor progression and treatment.

**Conclusion:**

This bibliometric study may aid researchers in the understanding of the knowledge base and research frontiers associated with FEN1. In addition, emerging hotspots for research can be used as the subjects of future studies.

## Background

Flap endonuclease 1 (FEN1) is a multifunctional and structure-specific endonuclease belonging to the Rad2 nuclease family and playing a significant role in DNA replication and repair [1]. FEN1 performs biological functions with loop endonuclease activity (FEN), 5′-3′ exonuclease activity (EXO) and gap endonuclease activity (GEN). It is involved in a variety of DNA metabolism pathways, including the maturation of Okazaki fragments, long-patch base excision repair, maintenance of telomere stability, apoptosis-induced DNA degradation and trinucleotide repeats and repair of stalled replication forks. Moreover, FEN1 is localized in the mitochondrial compartment. Deletion of yeast FEN1 homolog, Rad27 leads to severe depletion of the mitochondrial genome and defects in respiration, indicating that it has a significant role in maintaining mitochondrial DNA integrity [2]. FEN1 also interacts with other proteins to achieve optimal activity [3–5]. Based on these functions, FEN1 is important in maintaining genomic stability, as well as sustaining cell development and growth.

FEN1 has been associated with different types of cancers. Initial studies identified FEN1 as a tumor suppressor gene. FEN1 haploinsufficiency can promote tumor progression and higher FEN1 expression may result in reduced risk of malignant transformation in gastrointestinal cells [6–8]. However, later studies have shown roles of FEN1 in the initiation and promotion of tumor progression. FEN1 is overexpressed in ovarian, lung, prostate, colon, breast, stomach, kidney and pancreas cancer, and is associated with a poor prognosis [9–12]. FEN1 is identified to be involved in microhomology-mediated end-joining, and FEN1 inhibitor exhibits synthetic lethal in vitro, indicating that FEN1 inhibitors combined with PARP inhibitors could be possibly used in BRCA-deficient tumors [13]. These results revealed that FEN1 is a potential biomarker for certain tumors. As a result, further research will help us understand the occurrence and development of disease in relation to FEN1, as well as provide novel preventive and therapeutic strategies. In recent years, research related to the function of FEN 1 significantly evolved, but the molecular regulatory mechanisms require further investigation.

Bibliometrics is a novel approach used to evaluate the academic achievements in a research field through mathematical and statistical methods [14]. Numbers and variations of research published in various countries, institutions and laboratories will be analyzed to explore trends and hotspots in this field [15]. Bibliometrics analysis explores the intellectual base and research frontiers by conceptualizing and visualizing data related to published literature. This is beneficial for researchers who can acquire knowledge structure and understand research hotspots, ultimately helping them identify novel research directions [16]. CiteSpace is an efficient tool used to visualize bibliometric analysis [17]. Currently, a bibliometric analysis on FEN1 research activity has not been interrogated or published. Here, bibliometrics and visual analysis of research focused on FEN1 from 2005 to 2019 were performed to explore the hotspots and frontiers with the goal of providing guidance for future research endeavors associated with FEN1.

## Methods

### Data sources and search strategies

Literature was retrieved from the Science Citation Index Expanded (SCI-expanded) of Web of Science Core Collection database on 1 July 2020. We collected data from 2005 to 2019, spanning a total of 15 years. Search keywords used to enter the database included FEN1 AND LANGUAGE: (English) AND DOCUMENT TYPES: (ARTICLE OR REVIEW). Data were directly downloaded from the database as secondary data without further animal experiments. Therefore, no ethical approval was required.

### Data collection

Full records and cited references for the publications retrieved from the WoSCC database were exported and converted to .txt format. Next, these data were imported into Citespace5.7 R1 for visualization analysis and mapping of publication characteristics.

### Statistical analysis

Graphpad Prism 8.4.0 was used to present the distribution of countries/regions, institutions, number of annual publications, number of citations and other characteristics. VOSviewer1.6.15 was then used to display the relationship between the countries and authors. Visual maps were generated using CiteSpace 5.7 R1.

## Results

### General information and annual publication outputs

**Fig. 1:**
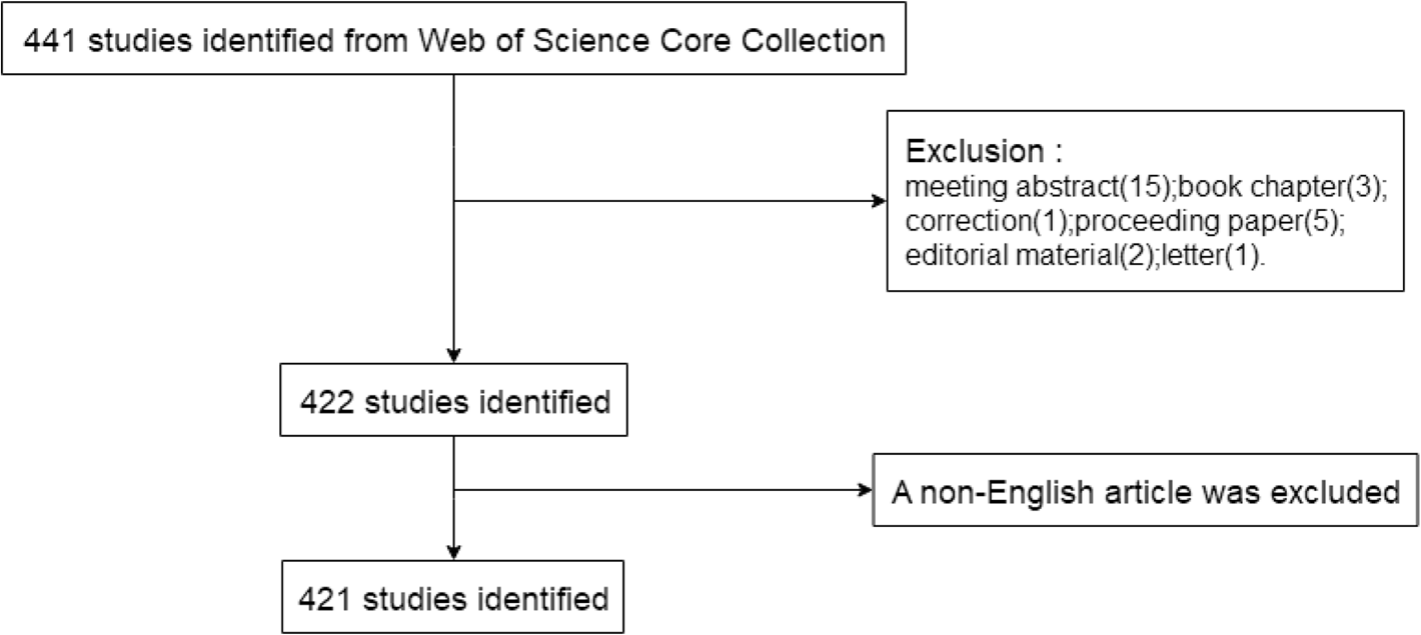
Flow chart of FEN1researches inclusion

A total of 441 studies were retrieved from the WoSCC database that were published within the period between 2005 to 2019. This included 395 research articles (89.6%), 27 review papers (6.1%), 15 meeting abstracts (3.4%), 3 book chapters (0.7%), 1 correction (0.2%), 5 proceeding papers (1.1%) and 2 editorial materials (0.2%). The search criteria produced 421 pieces of literature. The retrieval strategy of this study is depicted in Fig. 1.

**Fig. 2:**
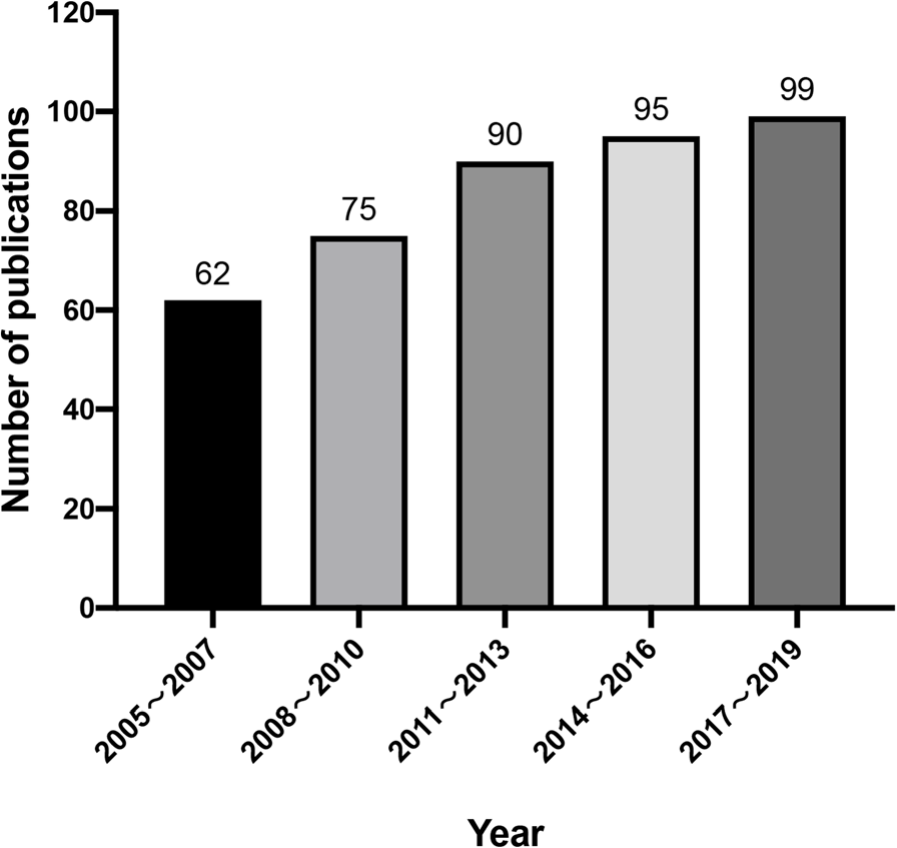
The number of publications in each time span on FEN1 research from 2005 to 2019

Included literature was cited a total of 13,396 times, with the average citation frequency being 31.82 for each publication and an H-index of 59. Using three years as a time span, the number of publications in each span was displayed as shown in Fig. 2. The number of FEN1 research articles published in each time span showed a steady upward trend, where from 2005 to 2013, the number of articles published rapidly increased. The total number of publications from 2011 to 2013 was nearly 1.5 times the number of publications generated from 2005 to 2007. After 2013, the number of publications remained relatively high but the growth rate slowed. This analysis revealed that FEN1 was intensively studied during the past 15 years and is still a popular research focus.

### Active countries and institutions

**Fig. 3:**
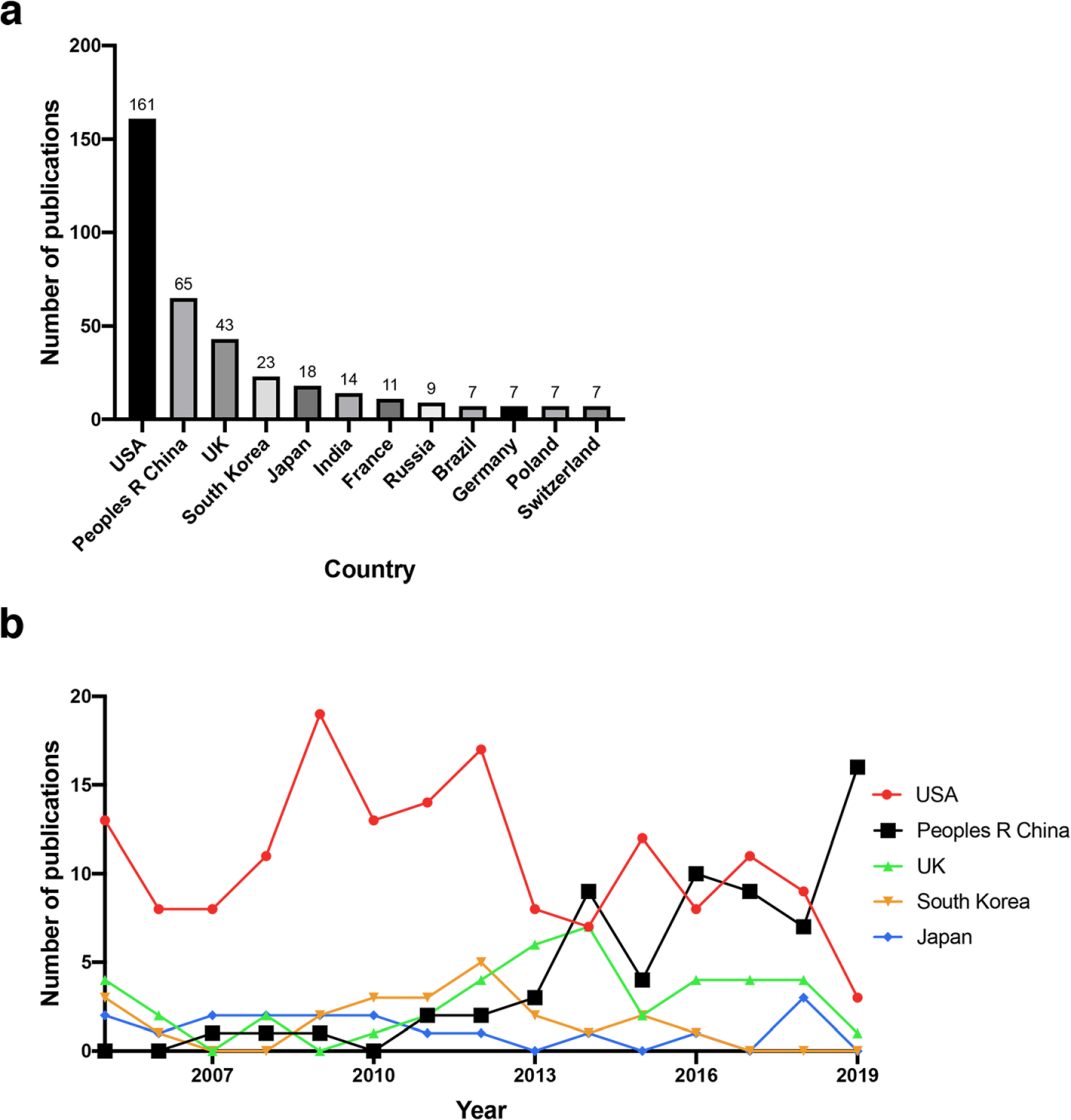
Analysis of countries. **a** Top 10 countries with largest number of publications on FEN1 research; and **b** The line chart of publications trend on FEN1 among different countries

The top 10 countries supporting publications on FEN1 in are listed in Fig. 3a. From 2005 to 2019, the United States had the most publications including 161 (38.24%) published articles and was followed by China (65, 15.44%), The United Kingdom of Great Britain and Northern Ireland (UK) (43, 10.21%), South Korea (23, 5.46%) and Japan (18, 4.28%). The United States and China contributed more than half of the total number of publications and were labeled as the two central research powers related to the FEN1 research field. The number of annual publications in these countries is shown in Fig. 3b. The peak FEN1 research period was shown to be from 2009 to 2012 in the United States. After 2013, research on FEN1 significantly increased in China, which has gradually become one of the most productive countries contributing to FEN1 research. In addition, FEN1 scientific outputs in the UK were also raised to a higher level in 2014.

**Fig. 4:**
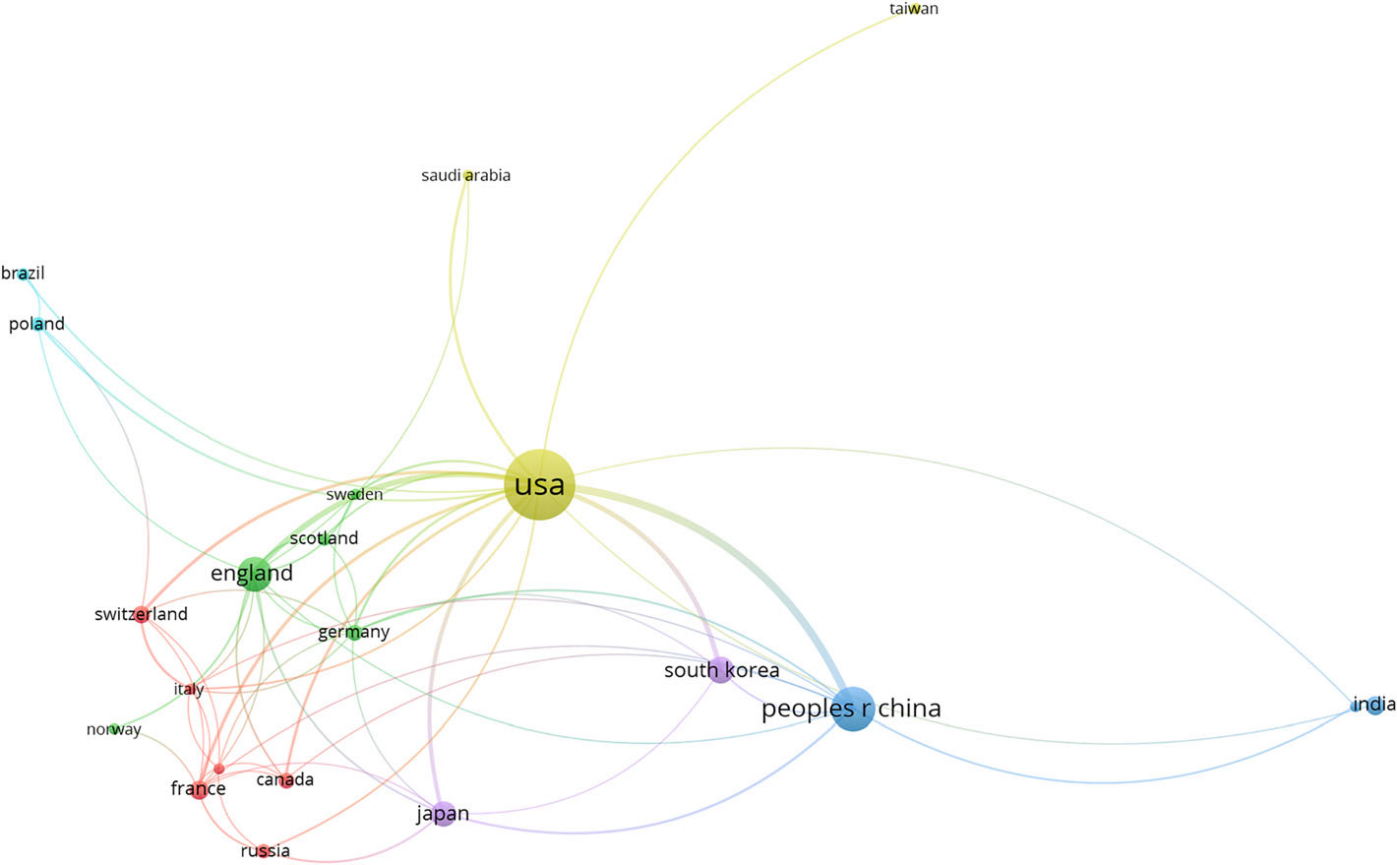
The network map of cooperation among different countries

Co-author analysis using VOSviewer displayed the cooperative relationship map among various countries. Thickness of the connection represented how close the collaborations were. As shown in Fig. 4, the United States and China, the top two productive countries, showed the closest cooperation. In addition, the United States had close collaborations with the UK and other countries, which demonstrated that this country emphasized research collaborations.

The top 10 institutions ranked by the number of publications on FEN1 are shown in Table 1. Beckman Research Institute of City of Hope produced the highest number of publications on FEN1 from 2005 to 2019 (57 publications), followed by National Institutes of Health (NIH USA), University of Rochester, Washington University (WUSTL) and NIH National Institute of Environmental Health Sciences. Seven of the top 10 productive institutions were located in the United States, which suggested that the United States has been one of the pioneers researching FEN1.

**Table 1:** The Top 10 institutions that contributed to the publications on FEN1 researches

No.	Institution	Number of Publications	Percentage (%)
1	Beckman Research Institute of City of Hope	57	13.54
2	National Institutes of Health (NIH USA)	45	10.69
3	University of Rochester	23	5.46
4	Washington University (WUSTL)	21	4.99
5	NIH National Institute of Environmental Health Sciences (NIEHS)	19	4.51
6	Zhejiang University	17	4.04
7	Harvard University	15	3.56
8	Korea Advanced Institute of Science Technology (KAIST)	15	3.56
9	Centre National De La Recherche Scientifique (CNRS)	14	3.33
10	California Institute of Technology	12	2.85

### Author analysis

**Fig. 5:**
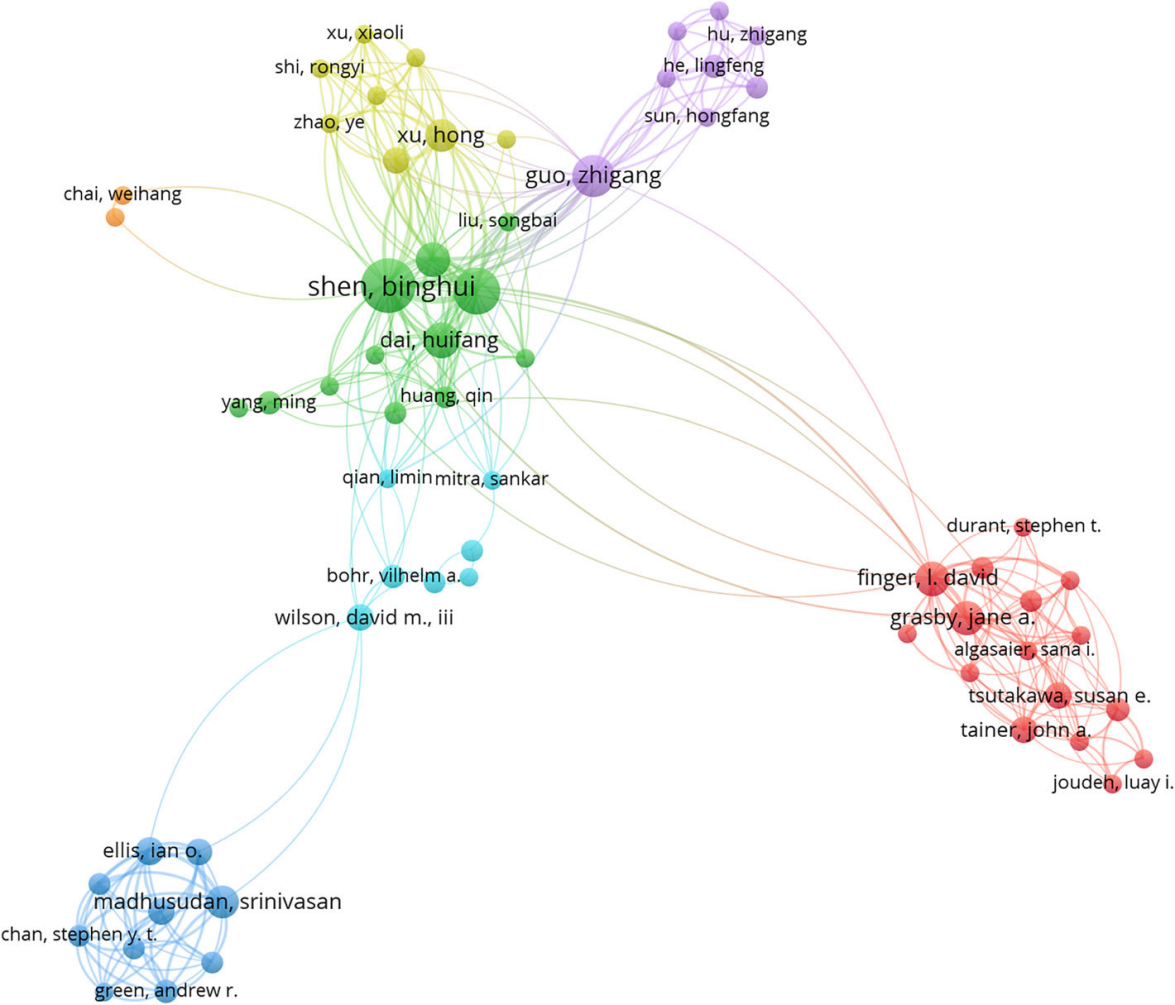
The network map of authors that conducted FEN1 researches

The top 10 authors who have published articles on FEN1 in the past 15 years are shown in Table 2. Both Shen BH and Zheng L contributed to more than 20 articles on FEN1. These authors are the most active and productive when it comes to the FEN1 scientific community. A network of collaborations among authors was conducted using VOSviewer as shown in Fig. 5. The visual mapping provides information related to potential collaborators or cooperative research teams and help researchers establish better cooperative relationships.

**Table 2:** The Top 10 authors that published articles on FEN1 researches

No.	Author	Number of Publications	Percentage (%)
1	Shen BH	25	5.94
2	Zheng L	22	5.23
3	Bambara Ra	19	4.51
4	Guo ZG	15	3.56
5	Seo YS	15	3.56
6	Campbell JL	12	2.85
7	Dai HF	11	2.61
8	Wilson SH	11	2.61
9	Finger LD	10	2.38
10	Grasby JA	10	2.38

### Journal analysis

In total, 181 journals published FEN1 related articles. Table 3 shows the top 10 journals containing the highest number of publications. Journal of Biological Chemistry had 48, accounting for 11.4% of total publications (IF = 4.238, 2020), making it including the highest number of FEN1 related studies. Nucleic Acids Research published 33 papers (7.8%), followed by DNA Repair, which published 21 and Plos One, which published 20 papers. These journals are considered to be the core journals in the field of FEN1 research.

**Table 3:** The Top 10 journals that published articles on FEN1 researches

Rank	Journal Title	Frequency *N* = 421	Percentage(%)	IF2020
1	JOURNAL OF BIOLOGICAL CHEMISTRY	48	11.4	4.238
2	NUCLEIC ACIDS RESEARCH	33	7.8	11.501
3	DNA REPAIR	21	5.0	3.339
4	PLOS ONE	20	4.8	2.74
5	PROCEEDINGS OF THE NATIONAL ACADEMY OF SCIENCES OF THE UNITED STATES OF AMERICA	8	1.9	9.412
6	BIOCHEMISTRY	7	1.7	2.865
7	CHEST	7	1.7	8.308
8	MOLECULAR CELL	7	1.7	15.584
9	ONCOGENE	7	1.7	7.971
10	EMBO JOURNAL	6	1.4	9.889

### Research area analysis

**Fig. 6:**
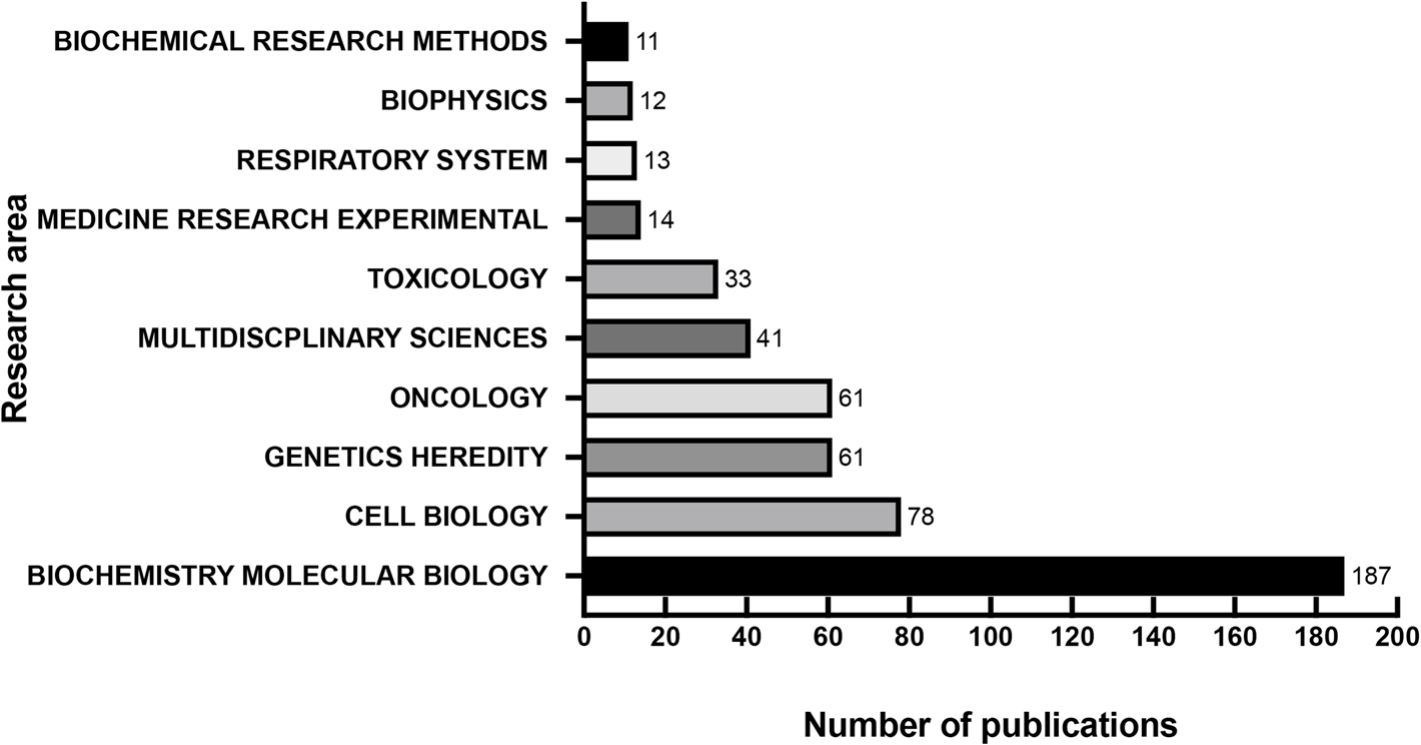
The top 10 research areas on FEN1 research

Figure 6 shows the top 10 research areas related to FEN1 research from 2005 to 2019. Biochemistry, molecular biology, cell biology, genetics heredity and oncology are the four areas where FEN1 were more studied.

### Co-cited references

**Fig. 7:**
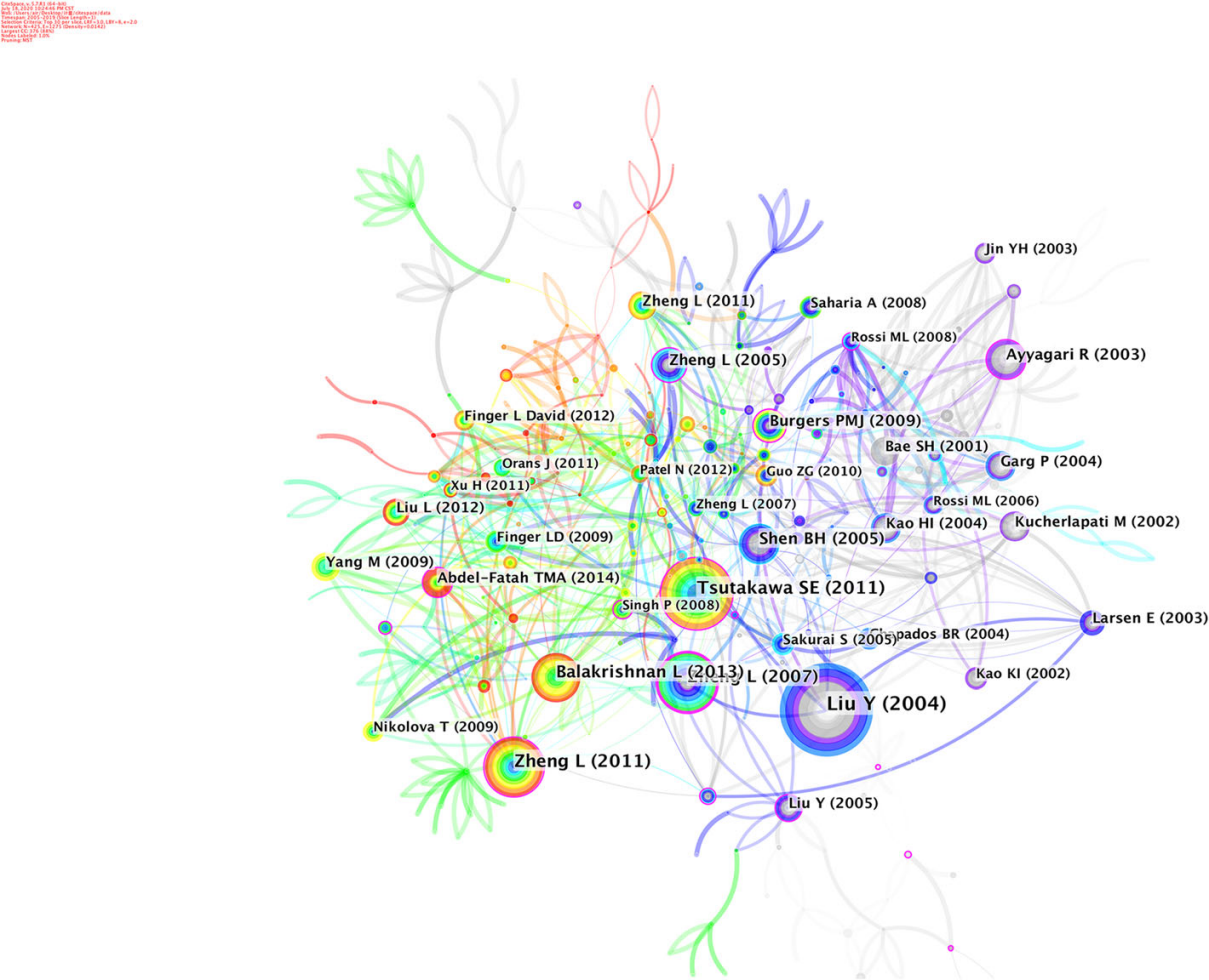
The analysis of Co-cited references: Co-citation network of references from publications on FEN1 research

A co-citation relationship is formed when two articles appear together in the references of the third citation literature. A total of 421 articles were visualized and analyzed using CiteSpace from January 2005 to December 2019. A time slice of 1 was selected to analyze the co-cited references. The network of co-cited references on FEN1 with high citation counts is displayed in Fig. 7. An intellectual base of this domain was generated using FEN1 papers with the most citations over the past 15 years. The top 10 references with the highest co-cited counts are summarized in Table 4. The highest cited paper was a review article published in ANNU REV BIOCHEM by Liu Y in 2004. The critical roles of FEN1 in DNA metabolism were highlighted in this study. The main content of this review included the discovery, structure, biochemical properties, substrate specificity, enzyme-substrate interaction and enzymatic mechanisms of FEN1, FEN1 tracking mechanisms, roles of FEN1 in Okazaki fragments maturation, DNA base excision repair, maintaining genome stability, repeat sequence expansions, interactions between FEN1 and other proteins and the expression of FEN1 and regulation of its activity [1]. Zheng L published several high cited studies on FEN1 and made considerable achievements this field. He discovered novel GEN activity of FEN1 in 2005, which was involved in processing stalled replication forks [18]. He also found FEN1 mutants and confirmed that FEN1 mutations are related to autoimmune diseases, chronic inflammation and cancer [19]. Among the articles published by Zheng L, the highest cited paper was a review article. In this article, the author introduced variable enzyme activities of FEN1 in RNA primer excision, base excision repair, fragmentation of DNA hairpin structure, apoptosis, DNA and other DNA metabolism pathways. Furthermore, localization, protein-protein interactions, post-translational modifications and other regulatory mechanisms of FEN1, as well asFEN1 mutations and correlation with cancer were summarized. Analysis of citation time demonstrated that this article has recently been highly cited. Another study that was highly cited recently, the structural and biochemical analysis were the focal points and the study revealed a uniform model of the FEN1 superfamily on substrate recognition and cleavage [20].

**Table 4:** The Top 10 co-cited references in FEN1 research

Rank	Frequency	Year	Author	Source	Co-cited Reference
1	76	2004	Liu Y	ANNU REV BIOCHEM	Flap endonuclease 1: a central component of DNA metabolism.
2	59	2011	Tsutakawa SE	CELL	Human Flap Endonuclease Structures, DNA Double-Base Flipping, and a Unified Understanding of the FEN1 Superfamily
3	49	2011	Zheng L	NUCLEIC ACIDS RES	Functional regulation of FEN1 nuclease and its link to cancer.
4	49	2007	Zheng L	NAT MED	Fen1 mutations result in autoimmunity, chronic inflammation and cancers.
5	42	2013	Balakrishnan L	ANNU REV BIOCHEM	Flap Endonuclease 1
6	37	2005	Shen BH	BIOESSAYS	Multiple but dissectible functions of FEN-1 nucleases in nucleic acid processing, genome stability and diseases.
7	32	2003	Ayyagari R	J BIOL CHEM	Okazaki fragment maturation in yeast. I. Distribution of functions between FEN1 AND DNA2.
8	30	2005	Zheng L	EMBO REP	Novel function of the flap endonuclease 1 complex in processing stalled DNA replication forks.
9	28	2009	Burgers PMJ	J BIOL CHEM	Polymerase dynamics at the eukaryotic DNA replication fork
10	27	2002	Kucherlapati M	P NATL ACAD SCI USA	Haploinsufficiency of Flap endonuclease (Fen1) leads to rapid tumor progression

In 2013, Balakrishnan L revealed that FEN1 is one of the most ancient proteins in the cell that has evolved to an efficient form and function and played an essential role in various DNA metabolism pathways [21]. In general, most of the highly cited articles related to FEN1 research are review papers focusing on the function and activity of FEN1.

**Fig. 8:**
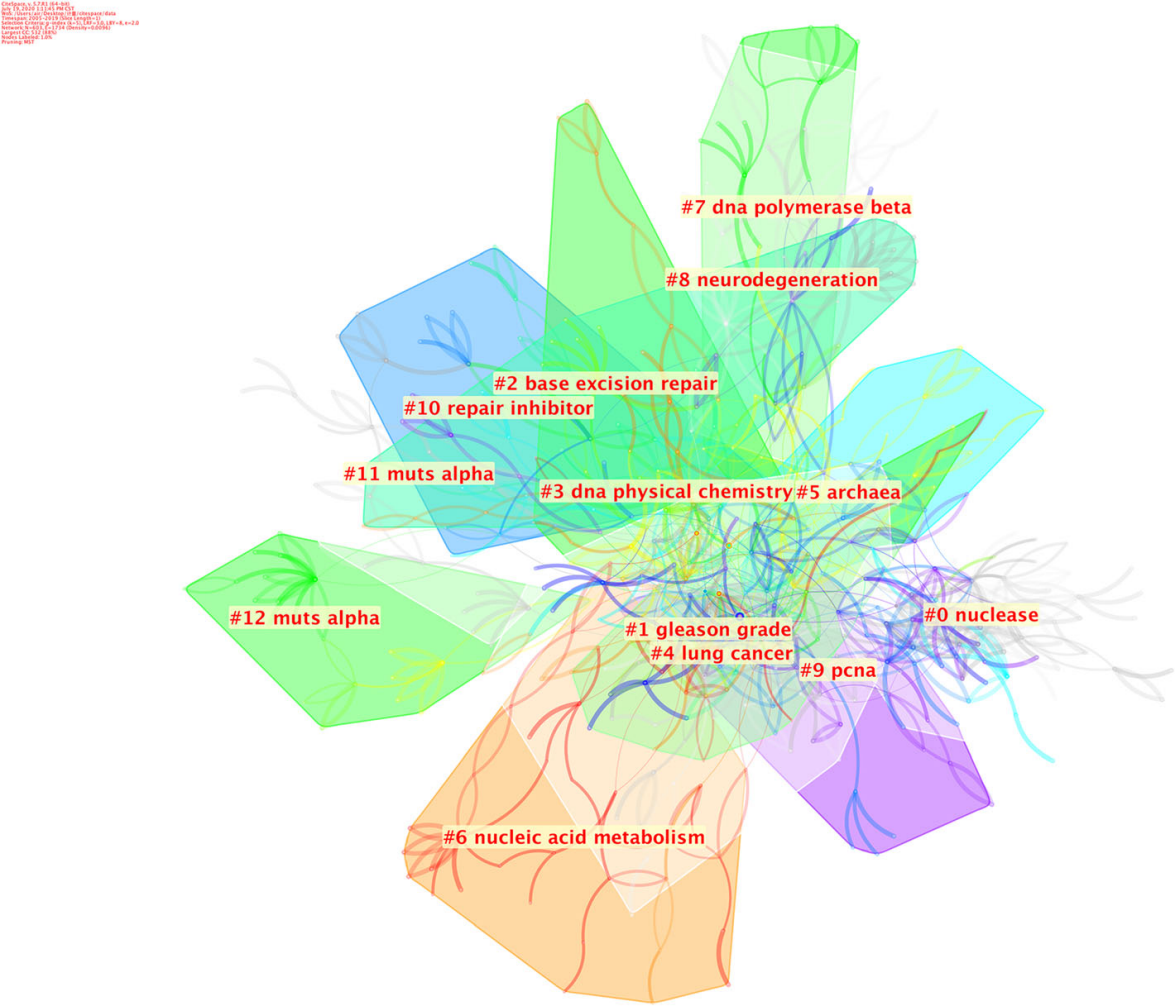
Cluster analysis of co-cited references

Based on the established citation network, cluster analysis was further performed to understand the knowledge structure in the FEN1research field (Fig. 8). Clusters with the largest capacity included nucleases, Gleason grade, base excision repair, DNA physical chemistry, lung cancer and archaea. This suggested that the evolution of the structure and function of FEN1 in lung cancer and prostate cancer may be the primary topics associated with FEN1 research.

### Keyword co-occurrence and burst

**Fig. 9:**
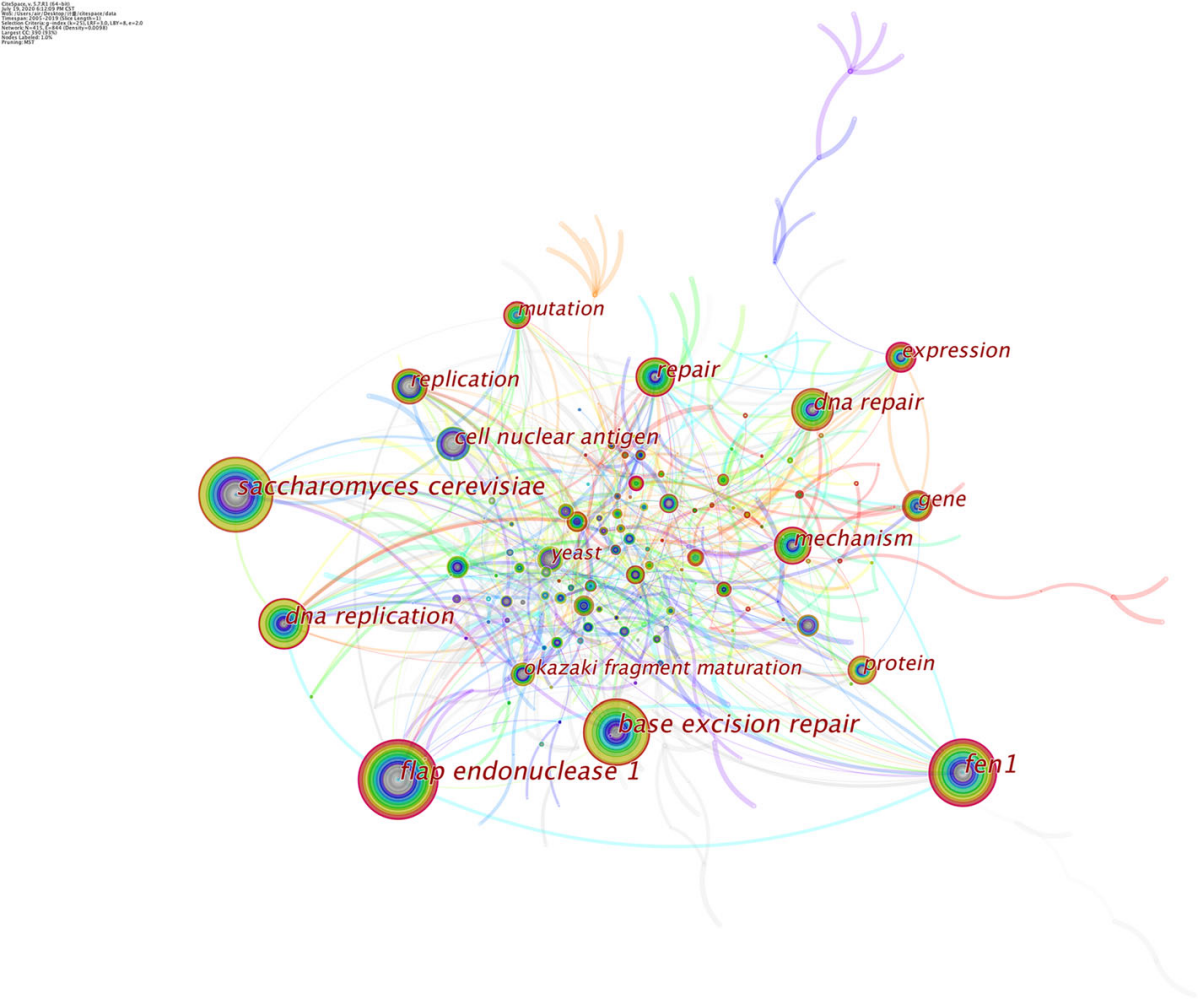
The network of keyword co-occurrence in FEN1 research

Keywords represent the core content of research. Keyword co-occurrence analysis detects the research hotspots in a certain field and burst keywords symbolize research frontiers over a period of time [22]. CiteSpace 5.7 R1 was used to establish a knowledge map of keyword co-occurrence (Fig. 9) and to identify the top 20 keywords based on frequency of FEN1 research from 2005 to 2019 (Table 5). The top keywords were “Flap endonuclease 1”, “*Saccharomyces cerevisiae*”, “Base excision repair”, “FEN1”, “DNA replication”, “DNA repair”, repair”, “mechanism”, “replication”, cell nuclear antigen”, “expression”, “protein”, “gene”, “mutation”, “Okazaki fragment maturation”, “yeast”, “binding”, “DNA polymerase beta”, “damage” and “replication fork”. As a result, research hotspots on FEN1 in the past 15 years can be summarized as FEN1 biology, FEN1 participation in replication and damage repair of DNA, the correlation between FEN1 and genetic mutations and interactions between FEN1 and other proteins.

**Table 5:** Top 20 keywords in terms of frequency in FEN1 research

Rank	Keyword	Frequency	Rank	Keyword	Frequency
1	Flap endonuclease 1	88	11	Expression	38
2	*Saccharomyces cerevisiae*	82	12	Protein	37
3	Base excision repair	81	13	Gene	35
4	FEN1	77	14	Mutation	33
5	DNA Replication	60	15	Okazaki fragment maturation	31
6	DNA Repair	50	16	Yeast	30
7	Repair	48	17	Binding	29
8	Mechanism	45	18	DNA polymerase beta	29
9	Replication	43	19	damage	27
10	Cell nuclear antigen	42	20	Replication fork	26

**Fig. 10:**
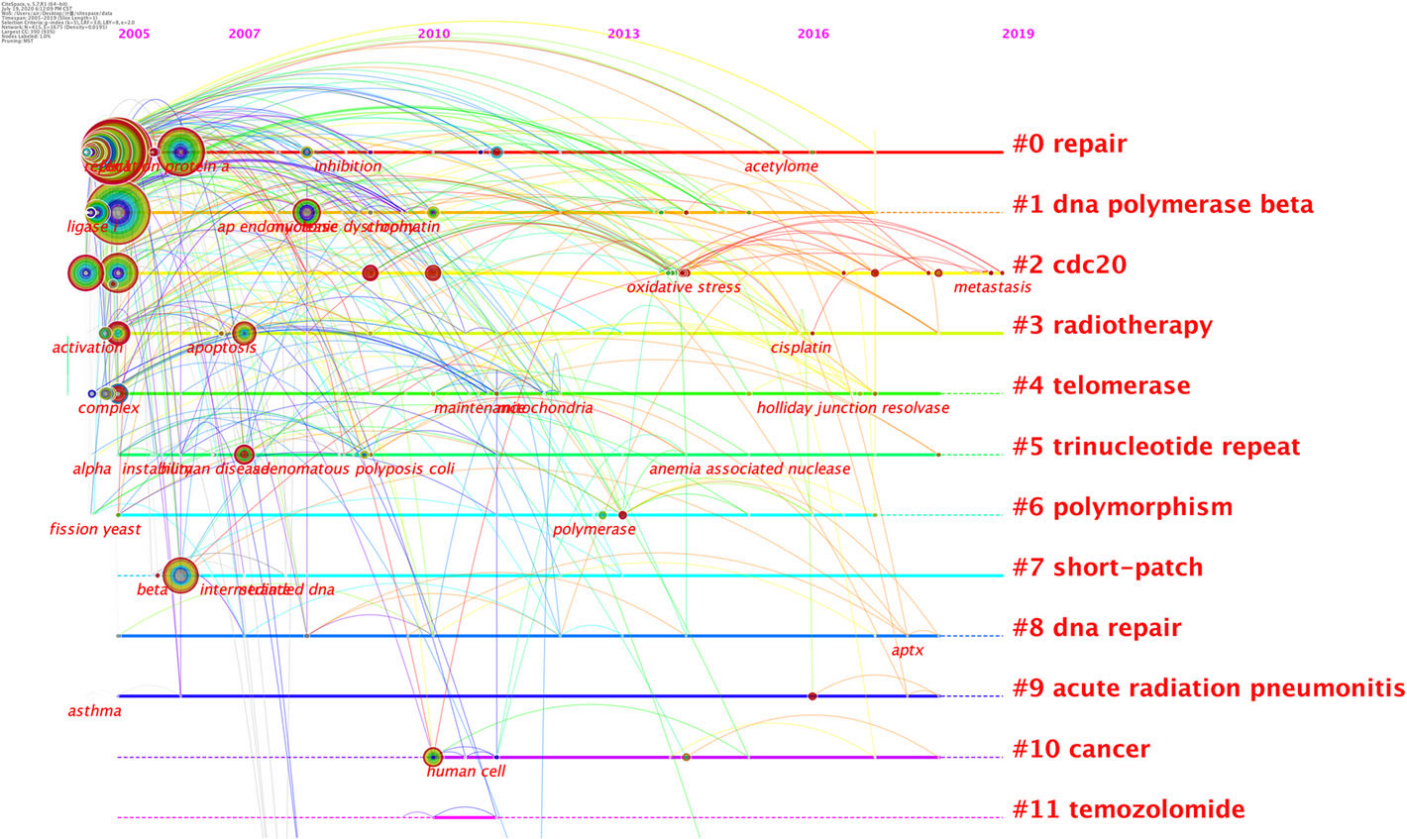
Timeline view of keywords cluster analysis in FEN1 research

By calculating the frequency of keywords in research on a certain topic burst keyword detection is conducted to identify research hotspots based on the growth rate of keywords. It can be used to observe emerging theories and themes and frontiers in a certain period of time [23]. Table 6 lists the keywords with the strongest citation bursts. As shown in Table 6 the keywords with strong bursts before 2014 were “cell nuclear antigen” “yeast” “*Escherichia coli*” “DNA polymerase delta” “replication protein A” “essential *in vivo*” “cleavage” “replication fork” “homologous recombination” “single stranded DNA” “genome stability” “nuclease” “polymorphism” “mutation”. While the burst keywords after 2014 mainly included “breast cancer” “oxidative stress” “strand break repair” “gene expression” “colorectal cancer” “phosphorylation” and “gastric cancer”. Meanwhile Citespace was used to perform a keyword cluster analysis and was displayed as a timeline view in Fig. 10. Combined with information in Fig. 10 we found that DNA damage repair was an existing research hotspot in the FEN1 field. The popular themes that emerged in recent years included oxidative stress hepatocellular carcinoma and resistance to cancer therapy.

**Table 6:**
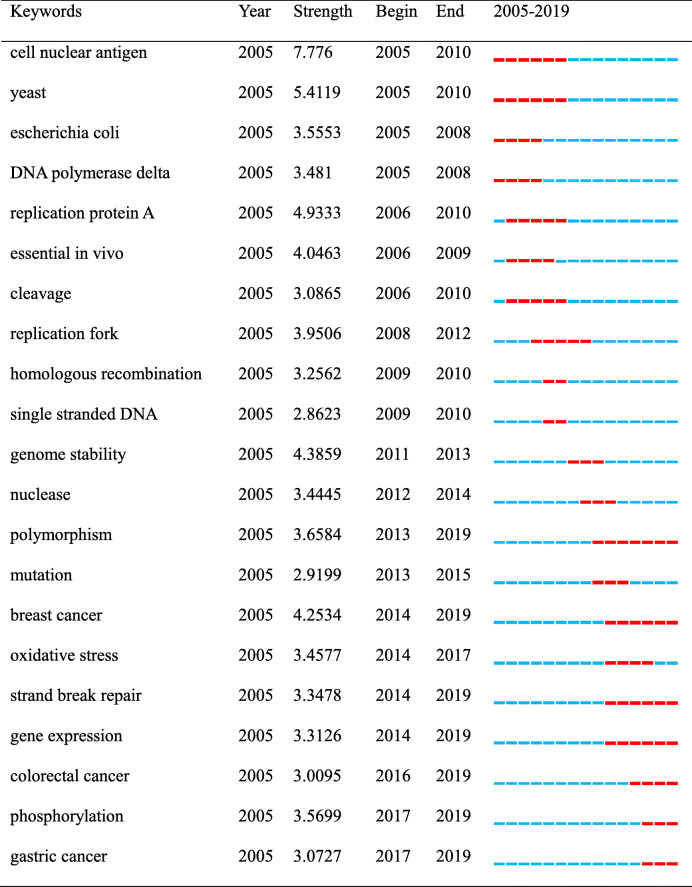
Top 21 keywords with the strongest citation bursts.

## Discussion

With the development of research on FEN1, potential values associated with clinical applications has attracted attention. FEN1 has become an emerging research area. Having better knowledge of FEN1 is crucial for future research. In this study, research conditions for FEN1 from 2005 to 2019 were analyzed for the first time mainly using the quantity of publications, co-citation analysis and keyword analysis.

In the past 15 years, trends in the number of articles published about “FEN1” significantly increased. This indicates that FEN1 is attractive to researchers and will be a valuable direction for future studies. From 2005 to 2019, the most productive country publishing on FEN1 was the United States and the institution with the most publications was present in this country. The United States mainly focused on FEN1 from 2009 to 2012. In China, FEN1 research was performed a bit later but developed quickly making it second in the number of FEN1 publications. Analysis of collaboration networks revealed collaborations between different countries, specifically between the United States and China. Effective collaborations between different countries should be further implemented to increase the robustness and number of studies related to FEN1. Shen BH was the most active author publishing articles on FEN1 and focused mainly on DNA replication, DNA repair and tumor biology. FEN1 was discovered and identified for the first time in his studies and he developed. The concept of structure specific endonucleases and greatly contributed to the FEN1 research field. Among the top 10 journals publishing on FEN1, six had an impact factor greater than 5. The number of articles published in these journals accounted for 16.2% of the total publications included in this study. In general, more than one-third of all literature on FEN1 was published in high impact journals. So far, the most relevant subjects of FEN1 included molecular biology, cell biology, genetics, heredity and oncology, which may continue to be the main subjects focused on in future studies.

Citespace was used to construct knowledge networks of FEN1. Co-cited analysis and keyword co-occurrence analysis showed that FEN1 research revolved around biological activity, mechanisms in DNA metabolism, interactions between FEN1 and other proteins and gene mutations. Burst detection suggested that keywords with strong burst over the past five years included breast cancer, oxidative stress, strand break repair, gene expression, colorectal cancer, phosphorylation and gastric cancer. Therefore, research frontier directions of FEN1 can be predicted as follows: mechanism research related to oxidative stress; research related to phosphorylation; mechanisms in occurrence, progression and therapy resistance in cancer. The results of this study provide an intellectual base and research frontiers for further studies performed for FEN1.

As shown here, FEN1 research mainly focused on biochemistry and molecular biology. The first homolog of FEN1 was identified in 1968. Eukaryotic FEN1 proteins consist of three domains including the N-terminal, intermediate and C-terminal domains. The N-terminal and intermediate domains show a high degree of homology among various species of bacteriophages, bacteria, eubacteria, archaebacteria, yeasts and mammals. FEN1 exhibits endonuclease activity, 5′-3′ exonuclease activity and gap endonuclease activity. The binding of FEN1 to its substrate is structure-specific rather than sequence-specific. Double flap DNA is the most suitable substrate for FEN1. FEN1 efficiently recognizes 5′ flap double-stranded DNA and 3’flap DNA significantly enhances its endonuclease activity. The nuclease activity of FEN1 requires the participation of divalent cations, such as Mg^2+^ and Mn^2+^. A pH of 8 is optimal for FEN1 enzyme activity in mammals [1]. Based on its nuclease activity, FEN1 performs important biological functions in Okazaki fragment maturation, long-patch base excision repair, processing of stalled replication fork and trinucleotide repeat sequence expansion. In addition, FEN1 maintains genomic stability by influencing the maintenance of microsatellite DNA and telomeres, non-homologous end joining, restricted homologous recombination, chromatin rearrangement, Holliday binding and double-stranded DNA break repair [20, 21, 24, 25]. FEN1 can also functionally and/or physically interact with other proteins to exert optimal activity. Proteins known to interact with FEN1 mainly include the following categories: (1) Proteins that help remove RNA primers during DNA replication, such as PCNA, DNA2, RPA, WRN, TRF2 and TERT; (2) DNA repair proteins, such as AP endonuclease 1 and β-pol; (3) Apoptotic proteins, such as CPS-6; and (4) Proteins post-translationally modifying FEN1, including p300, Cdk1-Cyclin A, Cdk2-Cyclin A and PRMT5 [1, 19]. The subcellular localization of FEN1 regulates its activity. Additionally, post-translational modifications including acetylation, methylation and phosphorylation influence the activity of FEN1 [6]. It has been confirmed that FEN1 Ser187 is phosphorylated by Cdk1-cyclin A or Cdk2/cyclin E complex in late S phase. Moreover, the phosphorylation of FEN1 caused by Cdk1-cyclin A in vitro can reduce endonuclease and exonuclease activities of FEN1 but does not affect DNA binding. However, phosphorylation of FEN1 can influence its interaction with PCNA [26]. According to the co-occurrence analysis, the discovery and mechanisms of proteins that interact with FEN1 may be a potential direction for future research. Furthermore, FEN1 is closely associated with oxidative stress damage repair. The balance between the oxidative and antioxidative system is disrupted when cells suffer from environmental stimulations in vivo or in vitro, such as by nitrogen oxide, calcium or pathogens. Reactive oxygen species (ROS) may be produced and accumulate, ultimately leading to oxidative stress. Oxidative stress can cause DNA damage in the form of DNA strand breaks, DNA site mutations, DNA double-strand aberrations, proto-oncogenes mutations and tumor suppressor genes mutations. At the same time, DNA damage caused by physical or chemical factors such as apurinic and apyrimidine, X-rays, ultraviolet rays and alkylating agents can also induce oxidative stress in vivo. Research show that oxidative stress is connected to mitochondrial diseases, family ataxia, Parkinson’s syndrome, Alzheimer’s disease and other neurological diseases, as well as tumors [27–29]. Base excision repair (BER) is the main method to repair DNA damage caused by oxidative stress [30]. The function of FEN1 in long-patch base excision repair appears to be particularly important in the repair of damage caused by oxidative stress. The mechanism of FEN1 involved in oxidative stress repair may also be an important topic for future research. FEN1 mutations can affect genomic stability and lead to the malignant transformation of cells. FEN1 mutations in *Escherichia coli* polymerase I (pol I) results in temperature sensitivity. It can also affect the maturation of Okazaki fragments. Defects in FEN1 will increase the rate of spontaneous mutations. Yeast genetic studies have shown that *S. pombe* rad2 and *S. cerevisiae* rad27 cause lethality when combined with a deletion of either the RAD51 or RAD52 genes [19].

Partial defects in FEN1 may also lead to the instability of single nucleotides or dinucleotide repeats, which is related to many diseases, especially tumors. In recent years, the research of FEN1 in oncology has increased, which has promoted the understanding of tumor occurrence, development, prevention and treatment. Oncology will remain a valuable topic for future research related to FEN1. Up-regulated expression of FEN1 is reported in various malignant tumor cells, including breast cancer, lung cancer, gastrointestinal tumors, hepatocellular carcinoma and cervical cancer. The abnormal expression of FEN1 in tumor cells is connected with the undermethylation of CpG islands in the FEN1 promoter region. In addition, overexpression of FEN1 is related to poor prognosis [9–11, 31–34]. Functional insufficiency of FEN1 haplotypes promote tumor progression [1]. FEN1 mutations or insufficient activity degrading apoptotic DNA may lead to chronic inflammation, immune diseases and tumor progression [6, 19]. Zhu H et al. found that AKT is a regulator of FEN1 activity in lung cancer cells. The continuous activation of AKT can phosphorylate the nuclear transcription factor NF-κB/p65. NF-κB/p65 directly binds to the promoter and induces FEN1 transcription. Upstream regulatory mechanisms of FEN1 in lung cancer was investigated in this study [35]. The latest study identified that small molecule inhibitors of FEN1 can significantly suppress the progression of homologous recombination (HR) deficient tumors, suggesting that FEN1 is a therapeutic target for HR deficient tumors [36]. Correlations between FEN1 and drug resistance were reported. Downregulation of FEN1 in glioma cells significantly increases its sensitivity to methylation drugs, such as methyl methanesulfonate and temozolomide [37]. Similarly, knock out of FEN1 enhances the sensitivity of gastric cancer cells to cisplatin [8]. As reported in breast cancer, FEN1 is related to the resistance of trastuzumab, tamoxifen, temozolomide, fluorouracil, cisplatin and other chemotherapeutics drugs [31, 38, 39]. Research published by Li JL suggested that inhibitors against FEN1 increase the sensitivity of cervical cancer cells to ionizing radiation therapy [32]. In osteosarcoma cells, the miR-193b/FEN1 axis activates autophagy and induces apoptosis, which are related to the sensitivity of epirubicin to chemotherapy [40]. Although many studies have been performed focusing on the association of FEN1 with tumor progression and drug resistance, the relevant mechanisms are not clear and further investigation is warranted. Above all, FEN1 inhibitor monotherapy or a combination with other drugs is promising for the treatment of tumors.

As far as we know, this is the first bibliometric analysis focusing on FEN1 research trends. The data in this study was retrieved from the WoSCC database and data analysis clearly displayed the research status of FEN1. However, some limitations were also present in this study. Aside from the WoSCC database, the PubMed, Scopus and Embase databases can also provide scientific literature. However, information offered by the WoSCC database is more detailed and the data covers the overwhelming majority of papers published in the FEN1 field. Considering the integrity and accuracy of the research process and results, articles and reviews published between 2005 and 2019 were selected. Other publications such as conference abstracts and comments were excluded and this exclusion may have led to missing some hotspots.

In conclusion, trends of FEN1 research from 2005 to 2019 were studied using bibliometric and visual analysis based on the WOSCC database. In the past 15 years, the number of publications on FEN1 steadily increased. The United States and China exerted an important influence on this domain. Effective cooperation between countries is beneficial for promoting FEN1 research. Research hotspots related to FEN1 included biological activity, mechanisms in DNA metabolism, interaction with other proteins and gene mutations. This study indicated frontiers of FEN1, which included oxidative stress, phosphorylation and tumor progression and therapy. Bibliometric analysis on FEN1 research is effective to aid researchers in identifying new direction, hotspots and frontiers related to FEN1 research.

## Data Availability

The datasets used and/or analyzed during the current study are available from the corresponding author on reasonable request.

## Abbreviations

FEN1: Flap endonuclease 1
WoSCC: Web of Science Core Collection
EXO: Exonuclease activity
GEN: Gap endonuclease activity
SCI-expanded: Science Citation Index Expanded
ROS: Reactive oxygen species
BER: Base excision repair
pol I: Polymerase I
HR: Homologous recombination
UK: The United Kingdom of Great Britain and Northern Ireland
